# *PC* Splice-Site Variant c.1825+5G>A Caused Intron Retention in a Patient With Pyruvate Carboxylase Deficiency: A Case Report

**DOI:** 10.3389/fped.2022.825515

**Published:** 2022-04-28

**Authors:** DongYing Tao, HuiQin Zhang, Jingmin Yang, HuanHong Niu, JingJing Zhang, Minghua Zeng, ShengQuan Cheng

**Affiliations:** ^1^Department of Pediatrics, XiJing Hospital, Fourth Military Medical University, Xi'an, China; ^2^State Key Laboratory of Genetic Engineering, School of Life Sciences, Fudan University, Shanghai, China; ^3^Medical Experiment and Training Center, Hanzhong Vocational and Technical College, Hanzhong, China

**Keywords:** pyruvate carboxylase deficiency, c.1825+5G>A, PC gene, splice-site variant, pathogenic variant

## Abstract

**Background:**

Pyruvate carboxylase deficiency (PCD; MIM#266150) is a rare autosomal recessive disorder characterized by a wide range of clinical features, including delayed neurodevelopment, elevated pyruvate levels, lactic acidosis, elevated ketone levels, and hyperammonemia. The pyruvate carboxylase (PC) gene was identified to be the disease-causing gene for PCD. A novel homozygous splice variant in the PC gene was identified in a Chinese boy, but the pathogenicity is still unclear. The objective of the present study was to determine the effect of this splice-site variant by reverse transcription analysis.

**Methods:**

We reported the clinical course of a 20-month-old Chinese pediatric patient who was diagnosed with PCD using whole-exome sequencing (WES). The effects of the variant on mRNA splicing were analyzed through the transcript analysis *in vivo*.

**Results:**

The results of metabolic blood and urine screening suggested PCD by employing tandem mass spectrometry. WES revealed a novel homozygous splice-site variant (c.1825+5G>A) in the PC gene. *in vivo* transcript analysis indicated that the splice-site variant caused the retention of 192 bp of the intron.

**Conclusion:**

Thus, c.1825+5G>A was established as a pathogenic variant, thereby enriching the mutational spectrum of the PC gene and providing a basis for the genetic diagnosis of PCD.

## Introduction

Pyruvate carboxylase deficiency (PCD; MIM# 266150) is a rare autosomal recessive inherited disorder with an estimated prevalence of 1:250,000. It results from the insufficient activity of pyruvate carboxylase (PC), a mitochondrial matrix enzyme, which converts pyruvate to oxaloacetatic acid, thereby facilitating gluconeogenesis and energy production (1, 2). Low activity of PC causes the accumulation of pyruvate, which is subsequently converted into lactate in the plasma by the enzyme lactate dehydrogenase. This eventually elevates plasma lactic acid levels. The decreased production of oxaloacetate decreases gluconeogenesis, thereby promoting hypoglycemia and preventing the liver from oxidizing pyruvate- and fatty acid-derived acetyl-CoA (2). This increases the acetyl-CoA pool, resulting in hepatic ketone body synthesis or ketoacidosis (2). It also affects the tricarboxylic acid (TCA) cycle and impairs the synthesis of aspartic acid, which consequently affects the urea cycle and the related biosynthetic pathways, thereby causing hyperammonemia. Another relevant role of PC activity involves astrocytes where the TCA cycle provides α-ketoglutarate as a precursor for the production of the neurotransmitter, glutamate (1–3).

Pyruvate carboxylase deficiency is associated with variants in the PC gene and is typically characterized by delayed development, recurrent seizures, elevated pyruvate levels, and lactic acidosis (3). PCD manifests in three main clinical forms: types A, B, and C. The infantile-onset form (type-A) manifests several months after birth; it is characterized by hypotonia, failure to thrive, delayed development, and lactic acidemia, followed by infection, diarrhea, and other symptoms, which eventually result in mortality during infancy or early childhood (4, 5). The neonatal-onset form (type-B) is usually characterized by severe lactic acidosis, hyperammonemia, and mortality within the first 3 months of life (6–8). The late-onset form (type C) presents with normal or mildly delayed neurological development and episodic metabolic acidosis (9, 10). To date, only 33 cases of PCD have been reported, and none of them are from the Chinese population. Here, we report the presence of a novel *PC* splice-site variant (c.1825+5G>A) in a 20-month-old Chinese boy and validate that the association of the variant is associated with abnormal *PC* mRNA processing, leading to intron retention.

## Materials and Methods

### Patient

The proband was a 20-month-old boy who was transferred to XiJing Hospital after presenting with delayed neurodevelopment and recurrent lactic acidosis. The patient was the first child of healthy consanguineous Chinese parents, with no significant family history. The child was born at full-term with normal weight, length, and head circumference. At the age of 3 months, the patient presented with bilateral eyelid clonus seizures, lasting from 30 s to 1 min, and was completely controlled by the oral administration of levetiracetam. At the age of 9 months, the patient presented with severe lactic acidosis with failure to thrive, tachypnea, and lethargy after 2 days of upper respiratory symptoms. Laboratory investigations during the acute episode revealed a plasma pH of 6.96 and the plasma levels of lactate, ammonia, and glucose, and alanine and citrulline levels to be 7.95 mmol/L, 93.77 μmol/L (limit: <40 mmol/L), 6.1 mmol/L, 715 μmol/L (normal range: 90–450 μmol/L), and 69.18 μ mol/L (normal range:5.5–45 μmol/L), respectively. The levels of lactate and ketones were also elevated in urine. MRI of the patient's brain revealed abnormal signal shadows in the white matter near the triangle of the bilateral lateral ventricles. Abdominal ultrasound indicated the absence of hepatomegaly. After intravenous administration of bicarbonate and hemodialysis, the patient's serum pH returned to normal. The patient subsequently presented with five additional episodes of lactate acidosis, which were treated with intensive fluid therapy; however, the patient's plasma lactic acid and ammonia levels remained elevated at 3.7–12 mmol/L and 36.25–93.77 μmol/L, respectively. The patient was slightly hypotonic, and his developmental milestones corresponded to those observed at 12 months of age. He could walk with support and pronounce approximately two words. Following diagnosis, the patient was administered biotin, aspartate, carnitine, thiamine, and citrate. At the age of 34 months, following one week of respiratory symptoms, the child developed severe metabolic acidosis. Despite treatment with dialysis, the patient's condition continued to deteriorate, with lactic acid levels increasing from 7.7 to 23.4 mmol/L, eventually followed by death.

### Whole-Exome Sequencing for Mutation Screening

To identify the gene responsible for the patient's clinical presentation, peripheral blood was collected from the patient and sent to Running Gene, Inc. (Beijing, China) for whole-exome sequencing (WES). Briefly, DNA was isolated and fragmented to build a DNA library by using the KAPA Library Preparation Kit (Illumina, Inc., San Diego, USA). Then, the library was sequenced using an Illumina HiSeq4000 platform (Illumina, San Diego, USA) using a 150-bp paired-end read according to the standard manual. The sequencing data was filtered and aligned with the human reference genome (GRCh37/hg19) by using the BWA Aligner (https://bio-bwa.sourceforge.net/), and variants were annotated by ANNOVAR (annovar.openbioinformatics.org/en/latest/). The exclusion of variants with minor allele frequency (MAF) >1% according to the gnomAD database (https://gnomad.broadinstitute.org/) and/or the 1000 Genomes Project database (https://www.genome.gov/27528684/1000-genomes-project), as well as *in silico* pathogenicity predictions for missense variants (SIFT: http://sift.jcvi.org, PolyPhen2: http://genetics.bwh.harvard.edu/pph2, MutationTaster: http://www.mutationtaster.org, FATHMM: http://fathmm.biocompute.org.uk, and CADD: http://cadd.gs.washington.edu) and splice-site variants (Varseak: https://varseak.bio/, splice AI: https://spliceailookup.broadinstitute.org/, dbscSNV_ADA and dbscSNV_RF: https://sites.google.com/site/jpopgen/dbNSFP). Variants were classified as: pathogenic, likely pathogenic, benign, likely benign and VUS, according to American College of Medical Genetics (ACMG) guidelines. A candidate causal gene mutation was identified and confirmed using Sanger sequencing.

### Transcript Analysis *in vivo*

Total RNA was extracted from the peripheral blood of the proband, his parents, and a healthy volunteer. Peripheral blood was collected in the PAXgene Blood RNA tube (BD Biosciences). RNA was isolated from the PAXgene Blood RNA kit (BD Biosciences) and then reverse-transcribed into cDNA by using PrimeScript RT Master Mix (Takara). The target sequence was amplified from cDNA by PCR. The primer of the amplification is forward GCCCAAAAGCTGTTGCACTAC and reverse AAGTCATCCCAGACATGGAATC. PCR products were separated on 1.5% agarose and sequenced with an ABI 3130 genetic analyzer (Applied Biosystems). Generated next-generation sequencing (NGS) library using the PCR product was sequenced by Illumina HiSeq4000 platform. PCR products were separated on 1.5% agarose and sequenced with an ABI 3730XL DNA analyzer (Applied Biosystems).

## Results

### Genetic Findings

**Figure 1:**
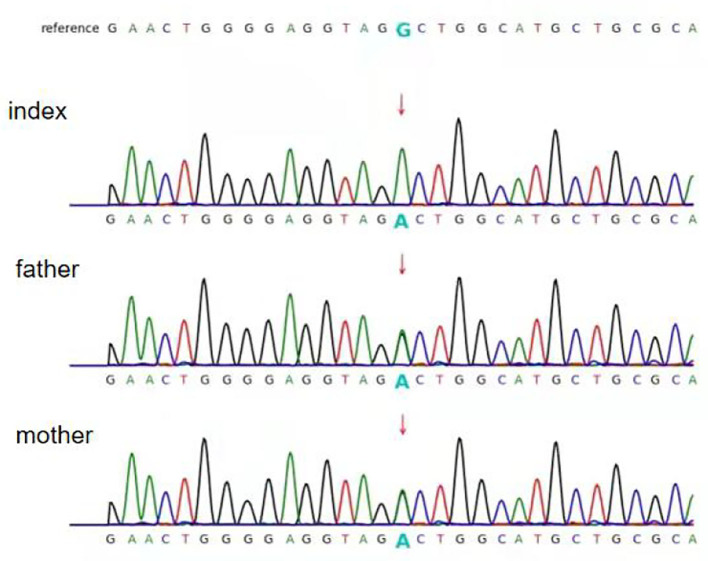
Partial sequence chromatograms of pyruvate carboxylase (PC). The red arrows represent the mutation site. Index: homozygous splice-site variant c. 1825+5G>A; father and mother: heterozygous splice-site variant c.1825+5G>A.

Exome sequence analysis revealed that the patient was homozygous for a specific variant (c.1825+5G>A) in the PC gene (NM_022172), and Sanger sequencing confirmed that both parents were heterozygous carriers of the variant (Figure 1). The variant was not found in either the Genome Aggregation Database (gnomAD) or 1000 Genomes database. According to the American College of Medical Genetics and Genomics (ACMG) guidelines (11), the pathogenicity of c.1825+5G>A was unclear. However, *in silico* prediction of the variant's effects, using four different algorithms (Varseek, splice AI, dbscSNV_ADA, and dbscSNV_RF), consistently predicted that the variant would affect splicing (Table 1).

**Table 1:** Summary of clinical findings of the proband.

	**Proband**			
Age of onset	3 months			
Clinical findings	Lactic acidosis	Developmental delay	Seziures	Recurrent metabolic neoacidosis
Laboratory	pH 6.96	Lactate 7.95	Ammonia 93.77 μmol/L	
	Plasma glucose 6.1	Alanine levels 715 μmol/L	Cit levels 69.18 μmol/L	
	Urinary organic acids:	elevated lactate and ketones		
MRI	Both abnormal signal shadows of the white matter near the triangle of bilateral lateral ventricles		
Genetic	PC:NM_022172 c.1825+5G>A			
*in silico* prediction	Varseek prediction level	4 [level range (1, 5)]		
	Splice AI	0.53 (range [0,1])		
	dbscSNV_ADA	0.9998 (range [0,1])		
	dbscSNV_RF	0.9499 (range [0,1])		
Time of diagnosis	At the age of 20-month-old			
Prognosis	At the age of 34 months, the child died of severe metabolic acidosis			

### *PC* MRNA Expression *in vivo*

**Figure 2:**
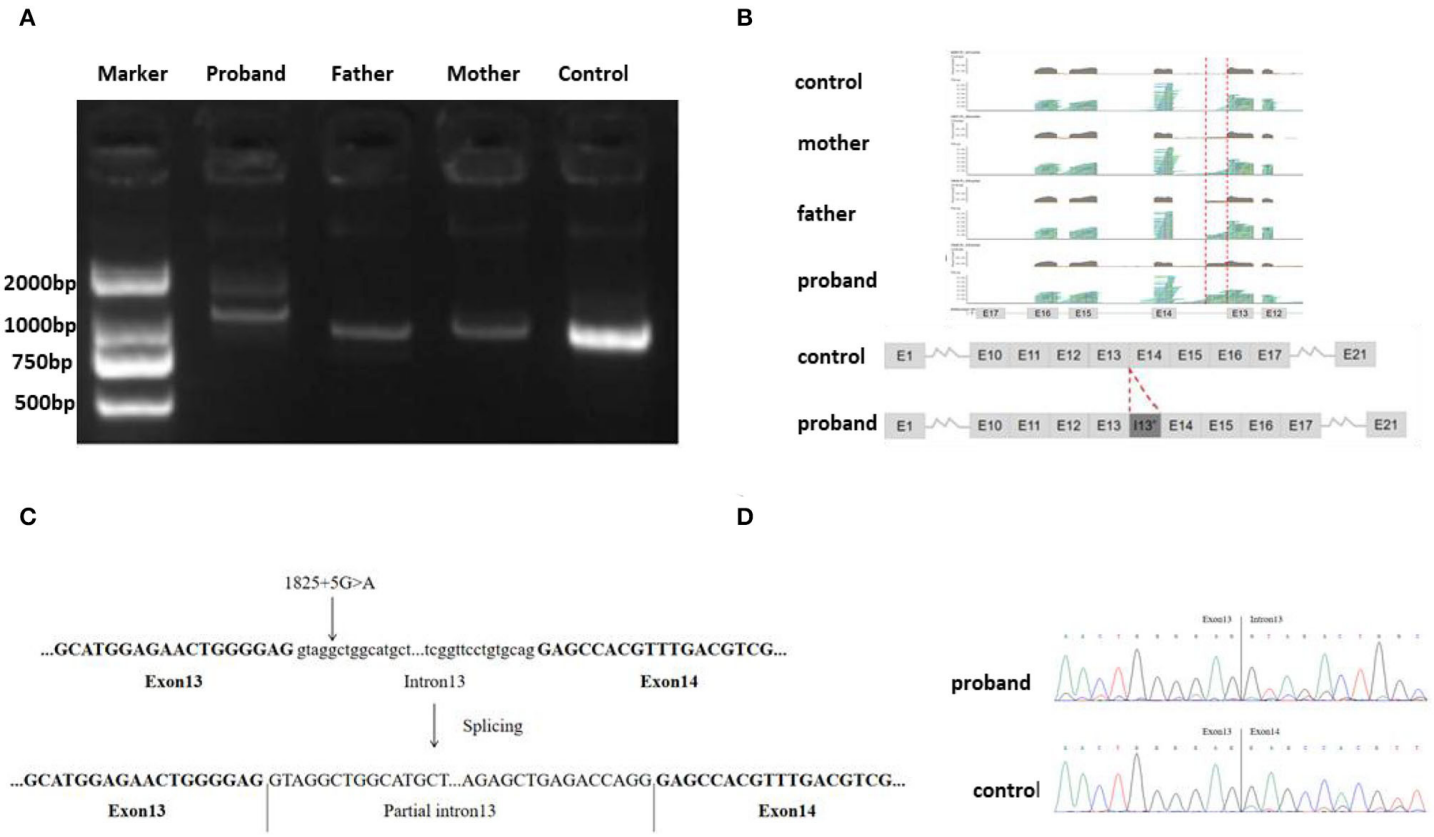
**(A)** Gel electrophoresis of RT-PCR fragments *in vivo* showed that the band of the proband (mut) was larger than that of control; **(B)** Next-generation sequencing (NGS) analysis of the complementary DNA (cDNA) showed that the proband cDNA retained a part of intron 13 (homozygous), and both parents retained parts of intron 13 (heterozygous); **(C)** A schematic of the PC gene showing the C, D position of the splice-site mutation. **(D)** Sanger sequencing indicated variant, c.1825+5G>A, results in the retention of the 192 bp base in intron 13 in the proband sample.

Agarose gel electrophoresis of RT-PCR-amplified genes showed that the band corresponding to that of the proband (mut) was larger than that of the control (wt) (Figure 2A). This indicates that the complementary DNA (cDNA) of the proband may have abnormal splicing. The cDNA amplification products of the proband, his parents, and healthy control were subjected to NGS. The results indicated that the proband cDNA retained a part of intron 13 (homozygous), and both the parents retained a part of intron 13 (heterozygous) (Figure 2B). Sanger sequencing indicated that the variant c.1825+5G>A caused aberrant splicing compared with that observed in the healthy control, resulting in the retention of the 192 bp intron 13 in the proband sample. Since the abnormal bands of his parents were too weak to be analyzed using the Sanger sequence, their sequencing results were similar to that of the control (Figures 2C,D). Eventually, the variant c.1825+5G>A was rated as pathogenic, based on the ACMG guidelines.

## Discussion

Pyruvate carboxylase deficiency is a rare neurometabolic disorder. Among the 34 cases reported to date, 10, including the proband of the present study, studies were born to consanguineous parents. The patient's symptoms first presented in the third month of his life and included delayed development, recurrent seizures, elevated pyruvate levels, lactic acidosis, hyperammonemia, and elevated ketone levels, which were consistent with the typical characteristics of type-A PCD (4). Among the eight patients that have been reported with type A PCD, two patients developed epilepsy, experiencing infantile spasms and tonic seizures. The symptoms were controlled by using a combination of multiple epilepsy drugs. However, the patient in the current study experienced a mild seizure, with the only form of attack of clonic seizure, which was well controlled with levetiracetam. Lactic acidemia and hyperammonemia seemed to respond to treatment with aspartate, biotin, carnitine, and thiamine. However, for the remaining conditions, anaplerotic therapy did not seem to affect disease progression. Despite medical interventions, episodes of severe acidosis with lethargy still occurred in the proband, following a respiratory tract infection. Eventually, the proband died during early childhood similar to the outcomes of other patients with type A PCD.

Here, we reported the identification, via WES analysis, of a novel *PC* splice-site variant. This variant was homozygous in the 20-month-old study patient and was inherited from close relative parents, who carried the heterozygous mutation at the same site. Notably, the variant was not found in the Genome Aggregation Database (gnomAD). *in silico* prediction of the variant's effects, using four different algorithms (Varseak, splice AI, dbscSNV_ADA, and dbscSNV_RF), consistently indicated that the variant affected PC mRNA splicing. Thus, the splice-site variant could explain the proband phenotype. To determine the effect of this PC splice-site variant, total RNA isolated from venous blood was transcribed into cDNA *in vivo*. Sanger sequencing analysis of the cDNA revealed that the splice-site variant could cause the retention of 192 bp of intron 13. The pathogenicity of the splice-site variant was classified as like-pathogenic (PS3+PM2+PP4), according to the ACMG guidelines (11). After comprehensive consideration of the clinical manifestations, genetic analysis, and cDNA sequencing results, the c.1825+5G>A variant was identified as the cause of the patient's PCD.

The PC gene maps to 11q13.2 and includes 20 coding exons as well as four non-coding codons (12). Biallelic variants in the PC gene are associated with PCD. According to the Human Genome Mutation Database and other studies, approximately 44 causative variants (Supplementary Table 1), including nonsense, missense, splicing, insertion/deletion, and frameshift mutations, have been identified in 33 PCD-affected individuals. Genotype/phenotype correlations in both the fatal PCD forms (types A and B) are unclear. Theoretically, the presence of at least one truncating mutation in the PC gene should generate more severe clinical phenotypes. The current proband carried biallelic mutations and presented as type A PCD. However, one case that was similar to our patient was homozygous for splice-site variant c.321+1G>T and was diagnosed with type-B PCD (13), while another case with biallelic missense mutation 506G>A also presented with type-B PCD (14). Thus, the correlation between genotypes and clinical phenotypes certainly requires further investigation.

For the differential diagnosis of PCD, practitioners should focus on identifying other hereditary metabolic diseases that cause hyperlactatemia and abnormal neurodevelopment, such as carbohydrate metabolic diseases, including type-I glycogen accumulation syndrome (GSD1) (15), pyruvate dehydrogenase complex deficiency (PDHCD) (16), hereditary fructose intolerance (17), and the fructose-1,6-bisphosphatase deficiency (1,6-FBD) (18). These disorders can be distinguished based on the fact that hypoglycemia and hepatomegaly are common in type I glycogen accumulation syndrome and fructose-1,6-bisphosphatase deficiency but not in PCD, where these effects are only seen in individuals with the type B form. In addition, while PDHD and PCD have identical clinical manifestations, blood ketone bodies are not detectable in PDHCD.

To the best of our knowledge, this is the first case to be reported among the Chinese population (4–9, 13, 14, 16, 19–21). The patient had 6-times higher metabolic acidosis, accompanied by increased levels of lactic acid, blood ammonia, and pyruvate. Due to insufficient knowledge, it was not accurately clearly diagnosed in the early stages. Further, patients manifesting similar clinical characteristics need to be evaluated for PCD, and *PC* tests can be further confirmed.

In summary, we report the identification of a novel splice-site variant (c.1825+5G>A) in the PC gene of a Chinese boy with type-A PCD. In addition, we confirmed the variant's pathogenicity, which involves an alteration in mRNA splicing. This report enriches the mutational spectrum of the PC gene and provides a basis for the genetic diagnosis of PCD.

## Data Availability Statement

The original contributions presented in the study are included in the article/Supplementary Material, further inquiries can be directed to the corresponding author.

## Ethics Statement

The studies involving human participants were reviewed and approved by the Medical Ethics Commilte of the first Afiliated Hospitat of the Air Force Medical University. The patients/participants provided their written informed consent to participate in this study. Written informed consent was obtained from the individual(s), and minor(s)' legal guardian/next of kin, for the publication of any potentially identifiable images or data included in this article.

## Author Contributions

DT, HN, and JZ was responsible for drafting the manuscript. DT did acquisition, analysis, and interpretation of data. HZ and JY designed and carried out the experiments. HN and JZ provided guidance and management for the patient. MZ made important comment for revision and polished the final version of the manuscript. SC was responsible for the revision of the manuscript for important intellectual content. All authors contributed to the article and approved the submitted version.

## Conflict of Interest

The authors declare that the research was conducted in the absence of any commercial or financial relationships that could be construed as a potential conflict of interest.

## Publisher's Note

All claims expressed in this article are solely those of the authors and do not necessarily represent those of their affiliated organizations, or those of the publisher, the editors and the reviewers. Any product that may be evaluated in this article, or claim that may be made by its manufacturer, is not guaranteed or endorsed by the publisher.
