# Supplementary material for: PC Splice-Site Variant c.1825+5G>A Caused Intron Retention in a Patient With Pyruvate Carboxylase Deficiency: A Case Report
Source: Front Pediatr. 2022 Apr 28;10:825515. doi: 10.3389/fped.2022.825515 (PMC9096210; doi:10.3389/fped.2022.825515)
Supplement: Supplementary file 1 [file Table_1.pdf]

Additional file 1: list of 44 causative variants have been described in 33 PCD-affected individuals globally

Clinical	Mutations	consanguineous	Major clinical findings	Reference
A	184C>T; 1892G>A; 2540C>T	/	Hypotonia, Mental retardation	13
A	c.467G>A	/	Lactic acidosis, Developmental delay	19
A	c. 808C>T； c. 1892G>A;	/	Lactic acidosis, Developmental delay	8
A	c.2668 G > T	/	Moderate lactic acidosis, Encephalopathy，polyuria，ketonuria	5
A	dup c.449_451 GGA	Yes	Severe lactic acidosis	4
A	c.1828G>A	/	Metabolic acidosis	4
A	c.1877 G>A/unknown	/	Bilateral clonic seizures, infantile spasms Metabolic acidosis	4
A	c. 2630A>G	Yes	Auditory neuropathy Spectrum disorder, infantile spasms and tonic-clonic seizures, Hypertonia	16
B	C.2491_2492delT/C.2473+2_2473+5delTAGG	/	Lactic acidosis seizures	20
B	C.1892G>A/C.2540C>T	/	Hypotonia, Rolling eye movements, Severe	13

			encephalopathy	
B	C.467G>A/C.496G>A	/	Metabolic acidosis	13
B	C.1892G>A/C.2492_2494delGT	/	Lactic acidosis,seizures	13
B	C.321+1G>T	/	Lactic acidosis,<br>Hyperammonemia	13
B	C.806G>A	/	Lactic acidosis	13
B	C.1748G>T/C.2876dupT	/	Lactic acidosis, Neonatal neurological distress	19
B	C.3392_3400del9/C.3363_3394dup32	/	Lactic acidosis, Neonatal neurological distress	19
B	C.370C>T	Yes	Hypothermia	6
B	C.615g>C/c.61G>C	Yes	Respiratory distress	6
B	C.2606G>A	/	Lactic acidosis	6
B	C.1369_29A>G/unknown	Yes	Lactic ketoacidosis	6
B	c.903+1G>A/unkown	/	Seizures	6
B	C.3288+1G>A/C.3463dupG	/	Respiratory distress；Hypoglycemia, liver affection	6
B	c.1240C>T/c.1240C>T	Yes	Hypotonia	6
B	C.616G>T/C.827A>C	/	Hypotonia	21
B	C.506G>A	Yes	Metabolic acidosis, hyperammonaemia, mild liver dysfunction	14
B	C.1154_1155delGC	Yes	Metabolic acidosis	14
B	C1023_1G>T/C.991A>G	/	Lactic acidosis, Neurological distress,Hepatic failure	8
B	C1131_1143del 12	Yes	Lactic acidosis and Hyperammonemia	7

C	c.1705A>G /c.3499-3500delCT	/	Episodes of metabolic acidosis with intercurrent illness	13
C	C.796T>G/c.2114C>A	/	Episodes of metabolic acidosis	13
C	c.796T > G/c.1267G > A	/	Metabolic acidosis	9
C	c.3242 A>G/c.751 + 4 A>G	/	Autism spectrum disorder, Metabolic acidosis	4
C	c.797 C>A/unknown	/	Metabolic acidosis	4
